# Prognosis of COVID‐19 patients using lab tests: A data mining approach

**DOI:** 10.1002/hsr2.1049

**Published:** 2023-01-08

**Authors:** Fariba Khounraz, Mahmood Khodadoost, Saeid Gholamzadeh, Rashed Pourhamidi, Tayebeh Baniasadi, Aida Jafarbigloo, Gohar Mohammadi, Mahnaz Ahmadi, Seyed Mohammad Ayyoubzadeh

**Affiliations:** ^1^ Administration and Resources Development Affairs Shahid Beheshti University of Medical Sciences Tehran Iran; ^2^ School of Traditional Medicine, Traditional Medicine & Materia Medical Research Center Shahid Beheshti University of Medical Sciences Tehran Iran; ^3^ Legal Medicine Research Center, Legal Medicine Organization Tehran Iran; ^4^ Non Communicable Diseases Research Center, Bam University of Medical Sciences Bam Iran; ^5^ Department of Health Information Technology, Faculty of Para‐Medicine Hormozgan University of Medical Sciences Bandar Abbas Iran; ^6^ Department of Health Information Technology, School of Allied Medical Sciences Shahid Beheshti University of Medical Sciences Tehran Iran; ^7^ Department of Pharmaceutics and Pharmaceutical Nanotechnology, School of Pharmacy Shahid Beheshti University of Medical Sciences Tehran Iran; ^8^ Department of Health Information Management, School of Allied Medical Sciences Tehran University of Medical Sciences Tehran Iran

**Keywords:** artificial intelligence, COVID‐19, data mining, Gradient Boosted Trees, machine learning

## Abstract

**Background:**

The rapid prevalence of coronavirus disease 2019 (COVID‐19) has caused a pandemic worldwide and affected the lives of millions. The potential fatality of the disease has led to global public health concerns. Apart from clinical practice, artificial intelligence (AI) has provided a new model for the early diagnosis and prediction of disease based on machine learning (ML) algorithms. In this study, we aimed to make a prediction model for the prognosis of COVID‐19 patients using data mining techniques.

**Methods:**

In this study, a data set was obtained from the intelligent management system repository of 19 hospitals at Shahid Beheshti University of Medical Sciences in Iran. All patients admitted had shown positive polymerase chain reaction (PCR) test results. They were hospitalized between February 19 and May 12 in 2020, which were investigated in this study. The extracted data set has 8621 data instances. The data include demographic information and results of 16 laboratory tests. In the first stage, preprocessing was performed on the data. Then, among 15 laboratory tests, four of them were selected. The models were created based on seven data mining algorithms, and finally, the performances of the models were compared with each other.

**Results:**

Based on our results, the Random Forest (RF) and Gradient Boosted Trees models were known as the most efficient methods, with the highest accuracy percentage of 86.45% and 84.80%, respectively. In contrast, the Decision Tree exhibited the least accuracy (75.43%) among the seven models.

**Conclusion:**

Data mining methods have the potential to be used for predicting outcomes of COVID‐19 patients with the use of lab tests and demographic features. After validating these methods, they could be implemented in clinical decision support systems for better management and providing care to severe COVID‐19 patients.

## 1 INTRODUCTION

The present coronavirus disease 2019 (COVID‐19) epidemic is an important public health issue on a global scale.^1^ Coronavirus disease (COVID‐19) has been caused by severe acute respiratory syndrome coronavirus 2 (SARS‐COV2), which points to the virus as the cause of the potentially fatal illness that is such a major global public health concern. In Wuhan, China, the first case of the disease was discovered in December 2019.^2^, ^3^ The World Health Organization (WHO) classified this illness as a pandemic on March 2020, due to its spread to other nations. COVID‐19 can spread one‐to‐one by respiratory transfer.^4^ Coughing, fever, and shortness of breath are some examples of COVID‐19 symptoms.^5^

Functional screening and diagnostic tools were imperative to control the outbreak of COVID‐19, isolation, precautions, and clinical management of patients. Artificial Intelligence (AI) assures a new platform for healthcare, aside from clinical procedures and treatments. Many various AI tools which made based on machine learning (ML) algorithms are used to analyze data and decision‐making processes.^1^ Medical practice has been gradually experiencing a change by AI. AI applications are growing into various fields which were before assumed to be only related to human researchers. This is due to the recent advancement in digitized data acquisition, computing infrastructure, and ML.^2^ It can even accurately distinguish between viral types of pneumonia, making it an effective screening tool using technologies such as AI and deep learning.^3^ Extracting knowledge from large data repositories made data mining an essential component in different fields of human life, and one of the most imperative parts is AI.^4^

Data mining as a subfield of AI can be defined as finding interesting patterns in data. The aim is to identify new, reliable, helpful, and comprehensible correlations and patterns in available data to use discovered patterns to explain the latest behavior or foresee the results.^5^, ^6^ Data mining techniques are extensively used in various healthcare applications such as patient outcome prediction, modeling of health outcomes, assessment of treatment efficacy, infection control, and ranking of hospitals.^7^

Research indicated that researchers should continue with the intuition they obtained, focusing on finding solutions to the problems of COVID‐19, and coming up with new developments. With the increasing focus on data mining and ML in the healthcare area, the proper environment can be provided for further advancement.^8^

Using data mining techniques regarding COVID‐19 issues could potentially be beneficial in overcoming these challenges. For example, accurately anticipating and diagnosing such viruses requires providing prediction systems, which can be challenging. Also, Identifying epidemiologic risks in advance will improve the prediction, prevention, and detection of future global health risks that could be predicted by AI‐driven techniques.^9^ Historical data can be a valuable source of information for predictive models. Data mining could also forecast who gets acute respiratory distress syndrome (ARDS), an acute and serious result of COVID‐19, with historical data.^10^ In this regard, Pan et al. at Vulcan Hill Hospital in Wuhan, China, extracted data from the COVID‐19 intensive care unit (ICU) to develop, evaluate and validate various ML models for predicting the prognosis of COVID‐19 patients.^11^ The combination of image datasets and ML has also helped in the diagnosis of COVID‐19. In the study of Muhammad et al. by using X‐ray images of patients' chests and ML models, they were able to extract the image features of COVID‐19.^12^, ^13^ In another study, Gumaei et al. used time‐series data on the number of people with COVID‐19 worldwide. They tried to predict patients with the disease using different ML models.^14^ Research has also been done to develop drugs in this area. Using ML techniques, Jamshidi et al. in their studies were able to reach, a framework based on DL methods that have been presented to illustrate how AI can accelerate the process of drug development. This framework includes eight layers, which are responsible for identifying, analyzing, and predicting the drug's performances in different stages.^15^, ^16^ The use of ML models has advanced to the point that it is even used to measure the impact of human interventions on the disease outbreak. For example, Delen et al. used the Gradient Boosted Trees model as a data mining technique to analyze the effectiveness of social distance during the COVID‐19 outbreak.^17^ Several similar studies have been conducted all around the world. For example, Gong et al. constructed an effective model to identify and classify cases of high risk. In their study, 372 after admission patients with COVID‐19 were monitored for more than 15 days, and models were made according to baseline data from two groups, including the severe and nonsevere groups. They used a nomogram for predictions of severe illness and assessed its performance. Findings in the training cohort and validation cohort were (area under the curve (AUC): 0.912 [95% confidence interval (CI): 0.846−0.978], sensitivity 85.71%, specificity 87.58%), and (AUC: 0.853 [0.790−0.916], sensitivity 77.5%, specificity 78.4%) respectively.^18^ Based on 14 clinical variables, 6 prediction models for COVID‐19 diagnosis were developed utilizing 6 distinct data mining approaches (BayesNet, Logistic, IBk, CR, PART, and J48). The study investigated 114 past instances from the Taizhou hospital in China's Zhejiang Province. Their findings demonstrated that the C.R. meta‐classifier, with an accuracy of 84.21%, is the most reliable classifier to predict positive and negative COVID‐19 instances.^19^ During the Corona outbreak, one of the greatest challenges for humanity was the proper management and response to this disease.^20^ According to the literature, ML can be useful in COVID‐19 research, diagnosis, and prediction.^21^ Therefore, for quick and very effective prediction to diagnose COVID‐19 patients, two stages of feature selection and COVID‐19 diagnosis stage can be used.^19^ Success in combating such epidemics depends heavily on building an arsenal of platforms, methods, approaches, and tools that converge to achieve desired goals and make life more satisfying.^22^

Although, as mentioned, many studies have been performed using different ML models worldwide, there is still a need to develop and evaluate these models with other datasets.^17^ The motivation and contribution of this study are to develop predictive models to determine the outcome of COVID‐19 patients using ML methods with a novel feature selection algorithm. These models were aimed to be trained based on Iranian hospitals' data that could help clinicians to prognosis COVID‐19 patients by Lab tests.

Thus, this study aimed to propose a prediction model for the prognosis of COVID‐19 patients (will the patient survive or die) using data mining techniques based on an Iranian data set of COVID‐19 patients. Accordingly, different steps were organized for this research. Overall, the methods section includes phases of data set collection, preprocessing, feature selection, and modeling and evaluation. In Section 3, the main findings of this study are presented which included the result of features selection and evaluation of data mining models as well as comparative indicators and diagrams related to the built models. In Section 4, the results of comparing the findings of our study with similar studies are represented. Finally, after stating the study's limitations, general conclusions are mentioned along with suggestions for future research.

## 2 METHODS

**Figure 1:**
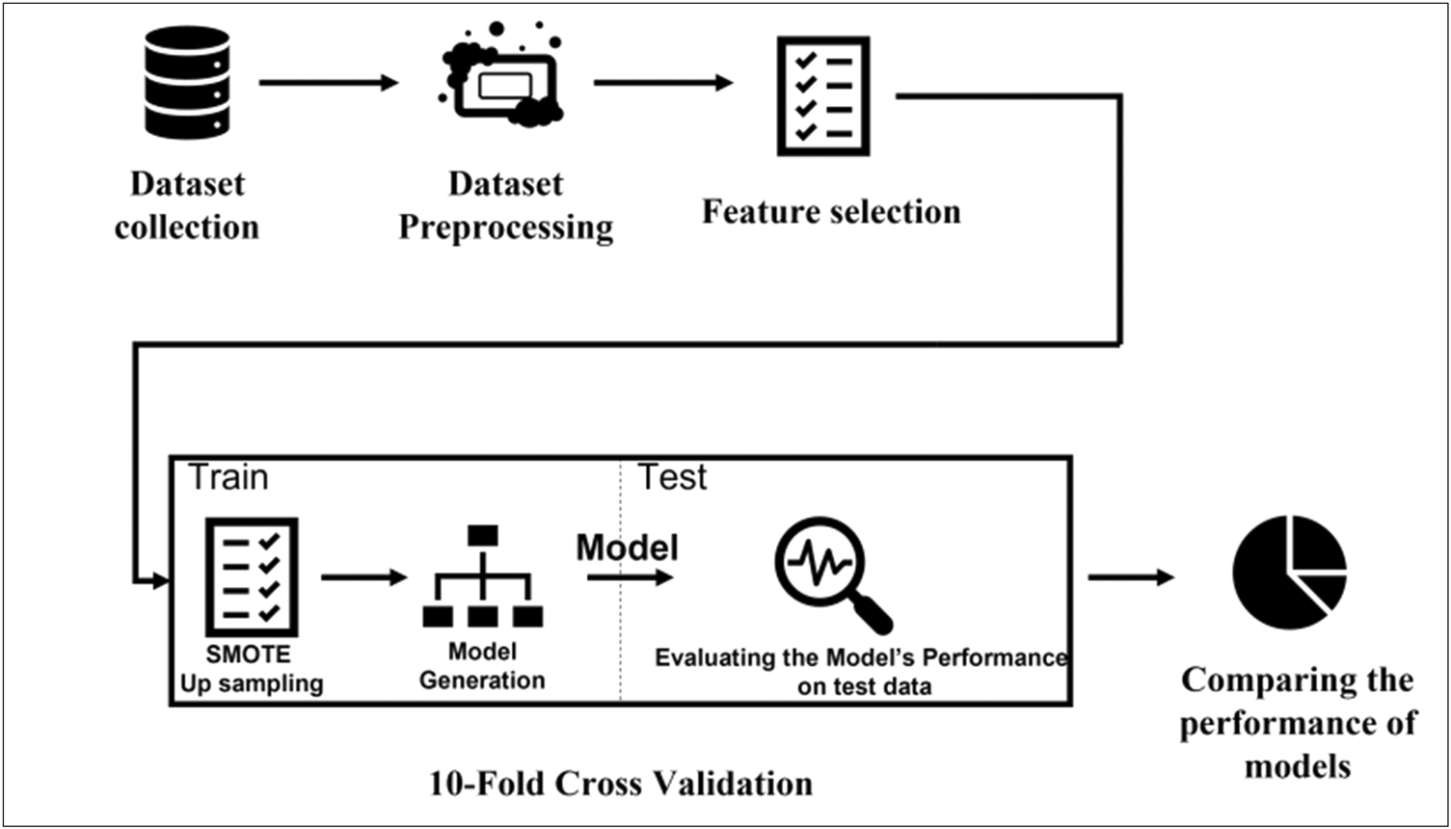
Steps of conducting the research

The methods used in this research are consistent with the related guidelines. The steps for conducting this research are represented in Figure 1. Overall, the method includes data set collection, data set preprocessing, feature selection, and modeling and evaluation which are described in the following sections.

### 2.1 Data set collection

The data set was obtained from the Hospital Intelligent Management (HIM) system repository, a comprehensive system containing patient data from 19 hospitals at Shahid Beheshti University of Medical Sciences in Iran.

All patients, as COVID‐19 cases, with a positive result of PCR test hospitalized between February 19 and May 12, 2020, were studied. The extracted data set has 8621 data instances. The data obtained include demographic features and results of 16 laboratory tests of patients. Demographic information includes gender, age, underlying disease, nation, and inpatient department. The laboratory tests include aspartate aminotransferase (AST), lactate dehydrogenase (LDH), lymphocytes, eosinophil (EOS), erythrocyte sedimentation rate (ESR), platelet count (PLT), alanine aminotransferase (ALT), hemoglobin, albumin calcium, magnesium, thyroid‐stimulating hormone (TSH), thyroglobulin (T.G.), fasting blood sugar (FBS), thyroxine (T4), triiodothyronine (T3), procalcitonin (PCT). The features data type is tabulated in Table 1.

**Table 1:** Feature data type

Feature name	Datatype
Gender	Binominal
Age	Numerical
Underlying disease	Nominal
Nation	Nominal
Inpatient department	Nominal
Aspartate aminotransferase (AST)	Numerical
Alanine aminotransferase (ALT)	Numerical
Lactate dehydrogenase (LDH)	Numerical
Lymphocytes	Numerical
Eosinophil (EOS)	Numerical
Erythrocyte sedimentation rate (ESR)	Numerical
Platelet count (PLT)	Numerical
Hemoglobin	Numerical
Albumin calcium	Numerical
Magnesium	Numerical
Thyroid‐stimulating hormone (TSH)	Numerical
Thyroglobulin (T.G.)	Numerical
Fasting blood sugar (FBS)	Numerical
Thyroxine (T4)	Numerical
Triiodothyronine (T3)	Numerical
Procalcitonin (PCT)	Numerical
Outcome (Label)	Binominal

### 2.2 Data set preprocessing

Preprocessing is a necessary process to produce an efficient classification model that impacts the performance of ML methods.^21^ In the first step, duplicate records were recognized and removed based on the national identification codes of patients to preprocess the data. The label (survived or dead) is transformed to binomial values in the data set.

In the next step, the data set containing the lab results of patients (Table 2) has been converted to a columnar data set with one patient per row and all test results and discharge states as columns.

**Table 2:** Sample records of the original laboratory test data set

Admission code	Test group	Test name	Result	Unit
3547430	ESR	ESR	35	mm/hr
3547430	AST	AST	25	U/L
3547430	AST	AST	24	U/L
3547430	ALT	ALT	23	U/L
3547430	ALT	ALT	20	U/L
3547440	PLT	PLT	439	×10^3^/µl
3547440	PLT	PLT	467	×10^3^/µl
3547440	PLT	PLT	511	×10^3^/µl
3547440	PLT	PLT	444	×10^3^/µl
3547440	PLT	PLT	355	×10^3^/µl

Abbreviations: AST, aspartate aminotransferase; ESR, erythrocyte sedimentation rate; PLT, platelet count.

Due to the differences in the lab tests for each patient, the resulting data set was sparse.

### 2.3 Feature selection

**Figure 2:**
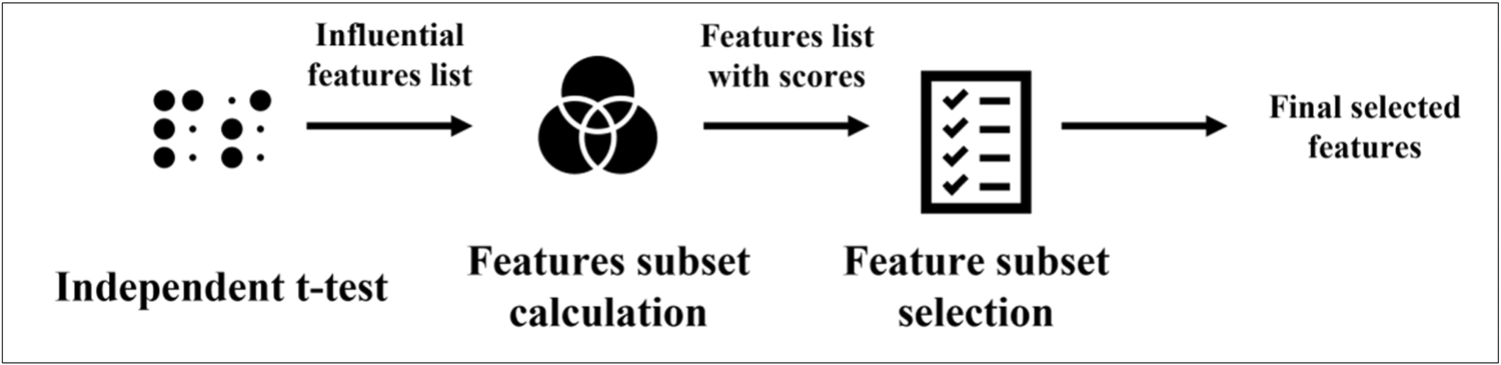
Feature selection process

The feature selection process is shown in Figure 2. The process includes an independent *t* test, features subset calculation, and feature subset selection that are described below.

#### 2.3.1 Independent *t* test

To choose the most influential factors in survival, the *p* values of the independent *t* test were calculated for each lab test in two groups (the survived patients' group and the dead patients' group). In this step, records with a missing value for that lab test are removed. The significant features were chosen to be passed to the next step (*p* value less than 0.05).

#### 2.3.2 Features subset calculation

**Figure 3:**
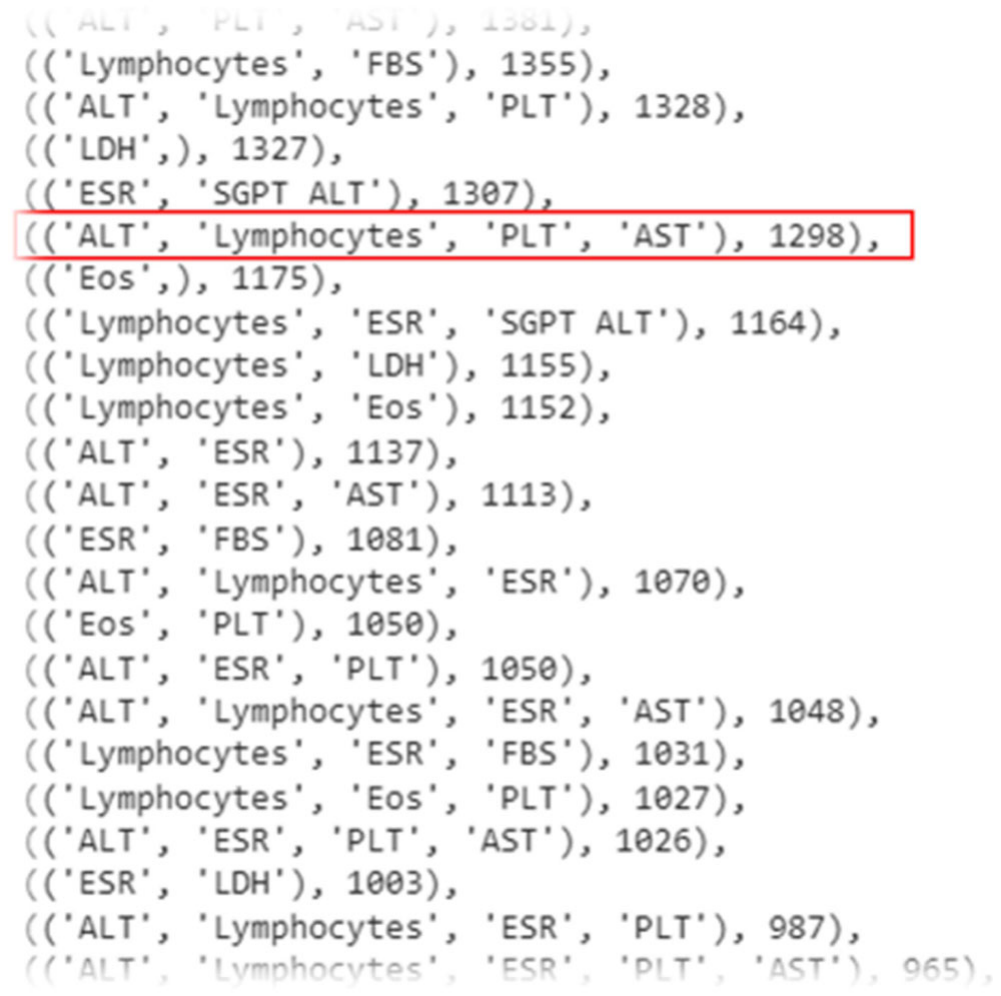
Sample features subset scores and the selected subset

After this step, to overcome the sparsity issue, we tried to generate a subset of features that have fewer missing values and more features. For this purpose, first, we listed all 32,767 possible subsets of the features list (15 features) and assigned a score to each feature subset. Each feature subset score is calculated with the count of the records having a value for all of the features in the subset. For example, in Figure 3, the record count having values of ALT, PLT, and AST features is equal to 1381.

### 2.4 Feature subset selection

The list of features subsets and scores related to each features subset is sorted, and the subset with the most features and a score of more than 1000 is chosen for the final data set (having more than 1000 nonmissing records for all features subsets with more features count; Figure 3).

#### 2.4.1 Modeling and evaluation

Logistic Regression, Gradient Boosted Trees, Naive Bayes, Decision Trees, Support Vector Machine, and Generalized Linear models are generated and evaluated using 10‐fold cross‐validation with Rapid Miner Studio software. The creating and assessing process of these models consists of feature selection, optimization of model parameters using the train data, and evaluation of the model using the test data, in the training and testing phases, respectively. Also, synthetic minority oversampling technique^23^ is applied for balancing the train data. This process divides the data set into 10 nonoverlapping folds. Each of the 10‐folds is given to be used in the test stage, while all other folds are used collectively in the training stage. A total of 10 models are fit and evaluated on the 10 hold‐out test sets, and the mean performance is reported.

#### 2.4.2 Logistic regression (LR)

LR is a kind of regression analysis in statistics used to predict the outcome of a definite‐dependent variable from a set of predictor or independent variables.^24^ The relationship between a categorical variable and dependent factors of any kind of categorical, continuous, or binary can be analyzed using LR.^25^ When the dependent variable has two values (0 and 1 or yes and no), it can be used, referring to binary logistic regression.^26^

#### 2.4.3 Naive Bayes (NB)

NB is a subdivision of Bayesian decision theory called naive as the formulation makes some naïve assumptions and can classify documents astoundingly well.^27^ NB is one of the simplest probabilistic classifiers.^28^, ^29^ The classifier simplifies the learning process by anticipating that features are independent of given classes.^28^ The resulting classifier is significantly prosperous in practice, even often competing with more sophisticated techniques.^26^

NB is efficient in several practical applications. Text classification and medical diagnosis are examples of such applications.^27^

#### 2.4.4 Support vector machine (SVM)

An SVM is used for analyzing data, discovering patterns in classification, and regression analysis.^24^ As a powerful tool for data classification, this model can classify two categories, classifies two categories which are pointed by assigning them to one of two disjoint half‐spaces, in the case of linear classifiers in the original input space or nonlinear classifiers, in the higher dimensional feature space.^26^ The larger space between the two classes, the better the model will be. Also, SVM works much better on datasets with many attributes.^24^

#### 2.4.5 Gradient boosted trees

Additional trees are combined strategically in the gradient boosting tree method by correcting mistakes that previous models made. Thus, it is more likely to increase the accuracy of prediction. Using Gradient Boosting of regression trees, it is possible to produce competitive, robust procedures that are also interpretable for regression and classification, especially suitable for mining less than clean data.^30^ Boosting algorithms are relatively easy to implement and allow for experimentation with various model designs. The GBMs have demonstrated significant progress in the practical applications and challenges of data mining and ML.^27^

#### 2.4.6 Decision tree (DT)

As a promising tool, a DT can predict response to data by using classification or regression and is one of the primary data mining methods. If the features are grouped, classification is used, and if data are continuous, regression is used. DT is constructed of a root node, leaf nodes, and branches. The evaluation of the data is possible by following the path from the root node to reach a leaf node.^24^ Two phases make a tree, the first is tree‐growing (building), and the second is tree‐pruning. In the first phase, the algorithm begins with the entire data set at the root node; the data set is splatted into subsets, which is repeated for the next steps (for each subset) until each member becomes sufficiently small. In the next phase (tree‐pruning), to boost the accuracy of the tree, the whole tree is cut back to avoid over‐fitting.^31^

#### 2.4.7 Generalized linear model (GLM)

The GLM provides a comprehensive and favored method for statistical analysis. In particular, the ability to predict can be valuable for the assessment of the practical importance of the predictors and to compare competing GLMs.^32^ These models are easy to interpret, and the methods are theoretically well understood and explained.^33^ GLM extends the concept of the linear regression model.^34^ The term GLM came from Nelder and Wedderburn^35^ and McCullagh and Nelder.^36^ They reported that if the distribution of *Y*, as a dependent variable, is a member of the exponential class, the GLM could be specified by two components, including the distribution of *Y* and the link function.^37^

#### 2.4.8 Random forest (RF)

As an ensemble learning method, RF or random decision forest could be used for tasks such as classification and regression. In this model, many decision trees are constructed at training time and outputting the class, whether classification or regression.^38^ The random forest algorithm was suggested for the first time by L. Breiman in 2001. This model could present a general‐purpose classification and regression method. This technique could have a suitable performance in problems where the samples are few related to the factors. It utilizes some randomized decision trees and classifies the data using the combination of results of the decision trees. Furthermore, it can easily be served to large‐scale problems, adapted to different ad hoc learning tasks, and returns variable importance measures.^39^ Although RF performs better than decision trees, its accuracy is less than the gradient‐boosted trees. Having said that, data characteristics can impact their performance.^40^

#### 2.4.9 Model evaluation

Also, evaluating the performance of similar steps without performing the feature selection phase is possible. Sensitivity and specificity, as two vital key factors, could determine the validity of a model.^41^ Thus, the accuracy, sensitivity, and specificity of these models have been evaluated using a using10‐fold cross‐validation method using Formulas 1‐3:

(1) $$\mathrm{Accuracy}=\frac{\mathrm{TP}+\mathrm{TN}}{\mathrm{TP}+\mathrm{FP}+\mathrm{FN}+\mathrm{TN}},$$

(2) $$\mathrm{Sensitivity}=\frac{\mathrm{TP}}{\mathrm{TP}+\mathrm{FN}},$$

(3) $$\mathrm{Specificity}=\frac{\mathrm{TN}}{\mathrm{FP}+\mathrm{TN}}.$$

## 3 RESULT

### 3.1 Feature selection

The Independent *t* test *p* values of lab tests are shown in Table 3.

**Table 3:** Independent *t* test *p* values of lab tests

Lab test	*p* value
ALT	0.0
AST	0.0
Albumin(Alb)	0.00044
ESR	0.0
Eos	0.00016
FBS	1e−05
Interleukin 6	0.01216
LDH	0.0
Lymphocytes	0.0
PLT	0.03728
Procalcitonin	1e−05
SGPT ALT	0.0
T4	0.00154
TSH	0.01758
Total Protein	0.00391

Abbreviations: ALT, alanine aminotransferase; AST, aspartate aminotransferase; ESR, erythrocyte sedimentation rate; FBS, fasting blood sugar; LDH, lactate dehydrogenase; PLT, platelet count; TSH, thyroid‐stimulating hormone.

Of these lab tests shown in Table 3, Lymphocytes, ESR, PLT, and AST features are selected based on the scores (the score of this combination of features was equal to 1298). Finally, these four lab tests along with eight demographic features were selected with 1461 records for model creation.

### 3.2 Modeling and evaluation

Seven data mining techniques were applied to the data set created from the previous step. The evaluated models include Logistic Regression, Gradient Boosted Trees, Naive Bayes, Decision Trees, Support Vector Machines, and Generalized Linear, Random Forest algorithms. Indicators of Accuracy, Sensitivity, Specificity, and AUC related to the built models can be seen in Table 4.

**Table 4:** Indicators of accuracy, sensitivity, specificity, and AUC related to the built models

Model	Accuracy (%)	Sensitivity (%)	Specificity (%)	AUC
Gradient Boosted trees	84.80	73.87	88.95	0.894
Random Forest	86.45	60.70	96.22	0.893
Support vector Machine	84.74	51.24	97.45	0.891
Naïve Bayes	85.21	55.72	96.41	0.880
Logistic Regression	85.56	65.18	93.29	0.877
Generalized linear model	83.09	43.29	98.21	0.865
Decision Tree	75.43	28.20	93.39	0.608

Abbreviation: AUC, area under the curve.

**Figure 4:**
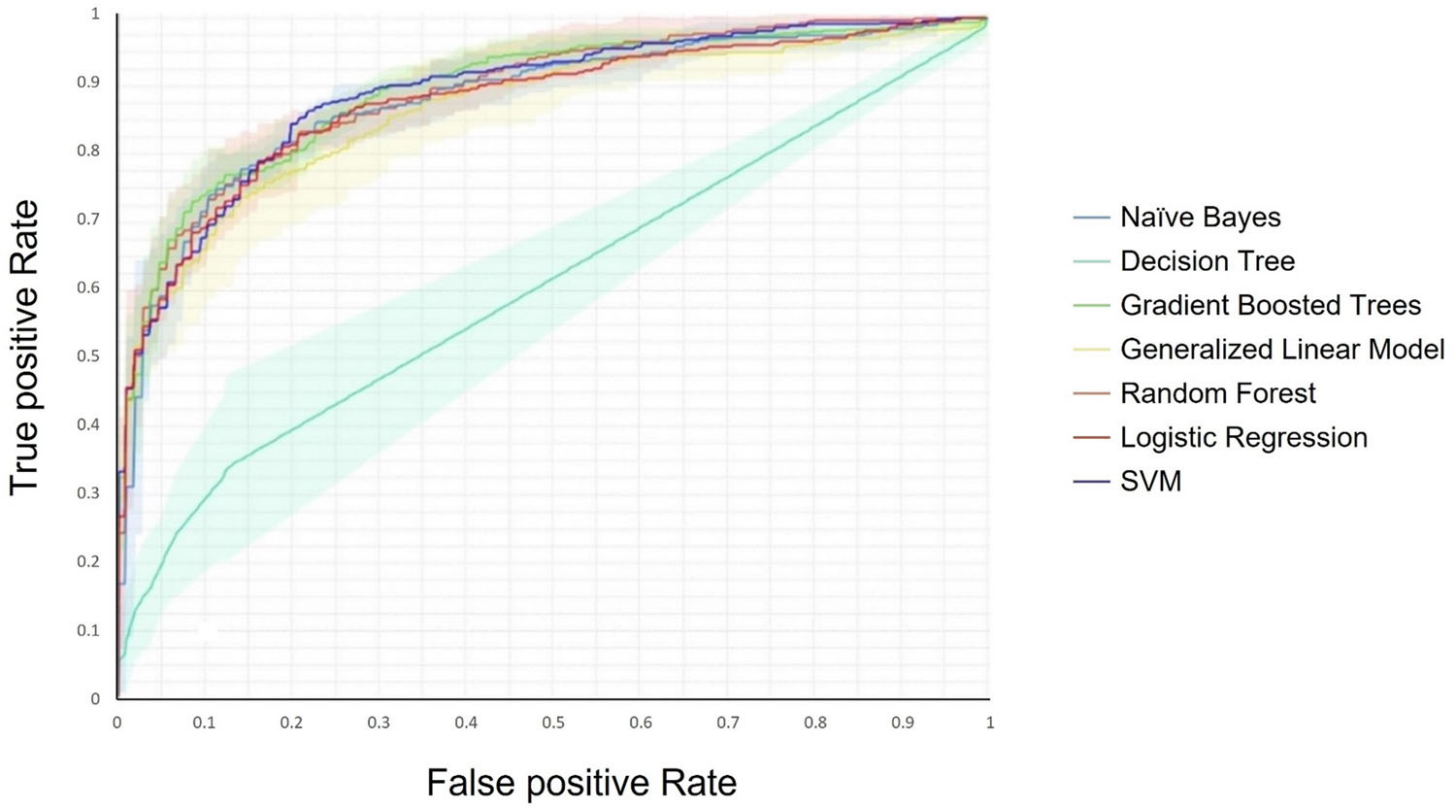
The comparison diagram of the ROC curve of the models

The Random Forest and Gradient Boosted trees models were the most efficient ones, with the highest accuracy percentage of 86.45% and 84.80%, respectively. In contrast, the Decision Tree had the least accuracy (75.43%) among the seven models. The comparison diagram of the receiver operating characteristic curve of the models can be seen in Figure 4.

## 4 DISCUSSION

This study examined the performances of classification models to predict COVID‐19 mortality based on seven models.

In the first step of this study, we concluded that among 15 laboratory tests, 4 of them have a relationship with patients who survived COVID‐19, which are: Lymphocytes, ESR, PLT, and AST. In the next step, data mining models were developed to predict COVID‐19 outcomes using the epidemiological data set of COVID‐19 patients from the Hospitals of Shahid Beheshti University of Medical Sciences. Logistic Regression, Gradient Boosted Trees, Naive Bayes, Decision Trees, Support Vector Machine, Generalized linear Model, and Random Forest algorithms were applied directly to the data set using Rapid Miner Studio. Based on laboratory tests and demographic features, our findings showed that the Random Forest and Gradient Boosted trees models had higher predictive accuracy than other models.

The growing importance of data mining in various sciences has led to the introduction of new models for this type of issue. Many studies have been done in medical sciences to predict the course of diseases using data mining models. A study by Che and others in 2016^42^ on the pediatric ICU data set for acute lung injury (ALI), using a method called interpretable mimic learning, which used the GBT model, showed that GBT could recognize important markers of mortality and ventilator‐free days prediction tasks. The 2020 study by Pan et al. was conducted on a database of patients with COVID‐19 in the ICU to compare conventional logistic regression methods and four ML algorithms. Finally, the eXtreme Gradient Boosting (XGBoost) model correctly predicted the risk of death with eight markers.^11^ The Gumaei et al. study was collected in 2020 using daily COVID‐19‐validated items. GBR had the best performance among all of the models.^14^

Delen et al., due to the excellent performance of GBT, this method was used to analyze the effectiveness of social distance during the outbreak of COVID‐19. This showed that about 47% of the changes in the rate of disease transmission could be explained by changes in the pattern of mobility resulting from the implementation of social distance policies in the studied countries.^17^

The GBT model is even used for early prediction of COVID‐19 using AI methods. Using a new Internet of Medical Things (IoMT) and GBT model, Yildirim et al. showed that the GBTs classifier has the best performance with 0.970 area under the curve (AUC) value for the diagnosis of COVID‐19 disease. This indicated the high performance of the GBT model in this study.^1^ In some data, such as genomic datasets, the GBT model is not necessarily the best model. In the study conducted by Akbulut et al. on Genomic Biomarkers of Metagenomic Next‐Generation Sequencing Data. It showed that the multilayer perceptron (MLP) model performed better than the GBT model.^2^, ^42^, ^43^

According to this study and previous studies, we believe that regarding COVID‐19 disease, the Gradient Boosted Trees model seems to have the best performance for laboratory tests such as ALT to predict the mortality of COVID‐19 patients. Although other models such as deep learning models showed an acceptable performance.^44^ Finally, due to the selection of the most effective tests for COVID‐19 mortality and the evaluation of the gradient‐enhanced tree model, this model will have the best performance in predicting mortality.

## 5 LIMITATION

One of the most critical limitations of this study is that the data were used only from hospitals under the supervision of Shahid Beheshti University of Medical Sciences, so the sample size was limited to the statistical population, which may show bias. However, all 19 hospitals make a large set, focusing on treatment and representing all patients with COVID‐19. The next limitation was the unequal distribution of patient laboratory tests, which affected data analysis.

In the future, according to what we have learned in this study, we can try to correct model defects and ML methods. We will also use a broader statistical sample that better represents the community.

## 6 CONCLUSION

Scientists and medical professionals have been working hard to find new ways to fight the infectious disease COVID‐19 and its various strains. Recently, ML and data mining methods have been proven to work successfully in healthcare for various purposes. Data mining methods, especially Gradient Boosted Trees and Random Forest models, can predict outcomes of COVID‐19 patients using lab tests and demographic features. In fact, we were able to predict patients at risk by using these models and selecting four lab tests from among 16 laboratory tests. These models should be evaluated in larger populations and different settings. After validating these methods, they can be used in the future for early diagnosis of critically ill patients and to prevent the increase in mortality in such pandemics.

## AUTHOR CONTRIBUTIONS

**Fariba Khounraz**: Conceptualization; data curation; writing – original draft. **Mahmood Khodadoost**: Conceptualization. **Saeid Gholamzadeh**: Conceptualization. **Rashed Pourhamidi**: Writing – original draft. **Tayebeh Baniasadi**: Supervision; writing – review & editing. **Aida Jafarbigloo**: Writing – original draft. **Gohar Mohammadi**: Conceptualization. **Mahnaz Ahmadi**: Writing – review & editing. **Seyed M. Ayyoubzadeh**: Conceptualization; data curation; formal analysis; writing – original draft.

## CONFLICT OF INTEREST

The authors declare no conflict of interest.

## TRANSPARENCY STATEMENT

The lead author Seyed Mohammad Ayyoubzadeh affirms that this manuscript is an honest, accurate, and transparent account of the study being reported; that no important aspects of the study have been omitted; and that any discrepancies from the study as planned (and, if relevant, registered) have been explained.

## ETHICS STATEMENT

This study design was reviewed and approved by the Ethics Committee of Shahid Beheshti University of Medical Sciences (IR.SBMU.RETECH.REC.1399.487).

## Supporting information

Supplementary information.

## Data Availability

Data available on request from the authors.
