# Supplementary material for: Prognosis of COVID‐19 patients using lab tests: A data mining approach
Source: Health Sci Rep. 2023 Jan 8;6(1):e1049. doi: 10.1002/hsr2.1049 (PMC9826741; doi:10.1002/hsr2.1049)
Supplement: Supplementary file 1 — Supplementary information. [file HSR2-6-e1049-s001.docx]

Appendix 1. Definitions

| Laboratory Test | Description |
| --- | --- |
| aspartate aminotransferase (AST) | “AST (aspartate aminotransferase) is an enzyme that is found mostly in the liver, but it's also in muscles and other organs in your body. When cells that contain AST are damaged, they release the AST into your blood. An AST blood test measures the amount of AST in your blood. The test is commonly used to help diagnose liver damage or disease. An AST blood test is often part of a routine blood screening to check the health of your liver.”  Ref: https://medlineplus.gov/lab-tests/ast-test/ |
| alanine aminotransferase (ALT) | “ALT stands for alanine transaminase. It is an enzyme found mostly in the liver. An ALT test measures the amount of ALT in the blood. Other names: Alanine aminotransferase (ALT), Serum Glutamic-Pyruvic Transaminase (SGPT), GPT “[1]  Ref: https://medlineplus.gov/lab-tests/alt-blood-test/ |
| lactate dehydrogenase (LDH) | “This test measures the level of lactate dehydrogenase (LDH), also known as lactic acid dehydrogenase, in your blood or sometimes in other body fluids. LDH is a type of protein, known as an enzyme. LDH plays an important role in making your body's energy. It is found in almost all the body's tissues, including those in the blood, heart, kidneys, brain, and lungs.” [1] https://medlineplus.gov/lab-tests/lactate-dehydrogenase-ldh-test/ |
| Lymphocytes | WBCs with anti-infection ability more common in viral ones [2] George-Gay B, Parker K. Understanding the complete  Ref:blood count with differential. J Perianesth Nurs. 2003;18(2):96-117. |
| eosinophil (EOS) | “An absolute eosinophil count is a blood test that measures the number of one type of white blood cells called eosinophils. Eosinophils become active when you have certain allergic diseases, infections, and other medical conditions.”  Ref:https://medlineplus.gov/ency/article/003649.htm#:~:text=An%20absolute%20eosinophil%20count%20is,infections%2C%20and%20other%20medical%20conditions. |
| erythrocyte sedimentation rate (ESR) | “An erythrocyte sedimentation rate (ESR) is a blood test that can show if there is inflammation in the body. Inflammation is the immune system's response to injury, infection, and many types of conditions, including immune system disorders, certain cancers, and blood disorders.”  Ref:https://medlineplus.gov/lab-tests/erythrocyte-sedimentation-rate-esr/#:~:text=An%20erythrocyte%20sedimentation%20rate%20(ESR)%20is%20a%20blood%20test%20that,Erythrocytes%20are%20red%20blood%20cells. |
| platelet count(PLT) | Platelet count in blood, these cells cause coagulation formation  Ref:George-Gay B, Parker K. Understanding the complete blood count with differential. J Perianesth Nurs. 2003;18(2):96-117. |
| Hemoglobin | Shows the amount of hemoglobin, responsible mainly for o2 transmission  Ref: George-Gay B, Parker K. Understanding the complete blood count with differential. J Perianesth Nurs. 2003;18(2):96-117. |
| Magnesium | “A magnesium blood test measures the amount of magnesium in the blood. Magnesium is a type of electrolyte. Electrolytes are electrically charged minerals that are responsible for many important functions and processes in the body.”  Ref:https://medlineplus.gov/lab-tests/magnesium-blood-test/ |
| thyroid-stimulating hormone(TSH) | “TSH stands for thyroid stimulating hormone. A TSH test is a blood test that measures this hormone. TSH levels that are too high or too low may be a sign of a thyroid problem.”  Ref:https://medlineplus.gov/lab-tests/tsh-thyroid-stimulating-hormone-test/ |
| thyroglobulin (T.G.) | “A thyroglobulin test measures the level of thyroglobulin in a sample of the blood. Thyroglobulin is a protein that thyroid makes.”  Ref:https://medlineplus.gov/lab-tests/thyroglobulin/ |
| fasting blood sugar(FBS) | “A blood glucose test measures the glucose levels in the blood.”  Ref:https://medlineplus.gov/lab-tests/blood-glucose-test/ |
| thyroxine (T4) | “A thyroxine test is a blood test that helps diagnose thyroid conditions. Thyroxine, also known as T4, is a type of thyroid hormone. A T4 test measures the level of T4 in your blood. Too much or too little T4 can be a sign of thyroid disease.”  Ref: https://medlineplus.gov/lab-tests/thyroxine-t4-test/ |
| triiodothyronine (T3) | “Triiodothyronine test measures the level of triiodothyronine (T3) in the blood. T3 is one of two major hormones made by thyroid,”  Ref:https://medlineplus.gov/lab-tests/triiodothyronine-t3-tests/ |
| procalcitonin (PCT) | “A procalcitonin test measures the level of procalcitonin in the blood. A high level of procalcitonin in your blood may be a sign of a serious infection or sepsis.”  Ref: https://medlineplus.gov/lab-tests/procalcitonin-test/ |
